# Postauricular linear basal cell carcinomas related to medical mask cords: a possible association

**DOI:** 10.55730/1300-0144.5720

**Published:** 2023-07-19

**Authors:** Bengü Nisa AKAY, Dilan KIZILIRMAK, Aylin Okçu HEPER

**Affiliations:** 1 Department of Dermatology, Faculty of Medicine, Ankara University, Turkiye; 2 Department of Pathology, Faculty of Medicine, Ankara University, Turkiye

**Keywords:** Linear basal cell carcinoma, trauma, pandemic, COVID-19

## Abstract

Linear basal cell carcinoma (BCC) is a distinct clinical morphological variant of BCC. Although it has been speculated that trauma and the Koebner phenomenon may be linked to linear BCC, the pathophysiology has not yet been shown. Herein, 5 cases of BCC were presented that developed in the postauricular region as a result of trauma caused by the cords of the medical face masks worn during the COVID-19 pandemic.

Basal cell carcinoma (BCC) is the most common skin cancer. Although ultraviolet exposure is the most significant etiological factor, others include ionizing radiation, chronic arsenic exposure, genetic predisposition, immunosuppression, scar tissue, and trauma. Although the pathogenetic mechanisms of phototoxicity, ionizing radiation, and genetic mutations have been well established in the development of BCC, the underlying mechanism of BCC after trauma and developing through scarring is restricted to theories [1, 2]. It has been speculated that persistent irritation and ulceration in scars on moving surfaces like joints may trigger neoplastic alterations and decreased cellular immunity in scar tissue may play a role in the development of BCC [3]. Herein, 5 cases of BCC were presented that developed in the postauricular region as a result of trauma caused by the cords of the medical face masks worn during the COVID-19 pandemic.

During end of the the second year of the COVID-19 pandemic, 5 patients were admitted to our department’s dermatoscopy unit with lesions behind their ears. The mean duration of the lesions was 8 ± 3.16 months, ranging from 8 to 12 months before admission. These 5 patients ranged in age from 57 to 83 (mean: 71.5 ± 10.03) years, and 2 (40%) were female. Histopathologically, the most common variant was the infiltrative type (n: 3), followed by superficial (n: 1) and basosquamous carcinoma (BSC) (n: 1). All of the tumors had a linear shape and were localized behind the ear, especially in areas exposed to the compression of the face mask cords. The characteristics of the patients are shown in the Table and Figure.

Linear BCC was first described by Lewis in 1985 [4]. It is a distinct clinical morphological variant of BCC. Although some authors have speculated that trauma and the Koebner phenomenon may be linked to linear BCC, the pathophysiology has not yet been shown [3, 4]. The postauricular region, where the cord of the masks generated friction, was the site of all of the lesions in our case series.

Considering the literature and small case series, it was noted that aggressive histological subtypes are more frequently observed in the linear presentation of BCC than in the general population; however, the most common linear type is nodular [5]. In line with the findings from the literature, 4 of the 5 lesions in our case series were aggressive subtypes of BCC, 3 were infiltrative, and 1 was BSC.

The presence of linear involvement in the lesions on the trail of the mask cord behind the ear, where pressure is felt more, suggests that prolonged trauma may contribute to the development of BCC. The linearity of the lesion may be connected to the inhibition of lateral spread brought on by reactive dermal fibrosis [5, 6]. This hypothesis might help to explain the linearity of lesions that form from the fibrotic dermis in the vicinity of radiation, trauma, or scars.

The majority of the patients in the present study had a history of numerous BCCs. In 2 cases, radiation had previously been applied to the scalp and neck for various reasons, and 1 patient was already receiving immunosuppressive medication. It should be clarified whether trauma facilitates tumor development in the population that already has risk factors for the development of BCC or whether it has a role in tumor development in the normal population.

In conclusion, it is critical to thoroughly examine the posterior of the ears while performing a dermatological examination on elderly patients to make sure that a more aggressive variant of BCC is not missed.

## Figures and Tables

**Figure:**
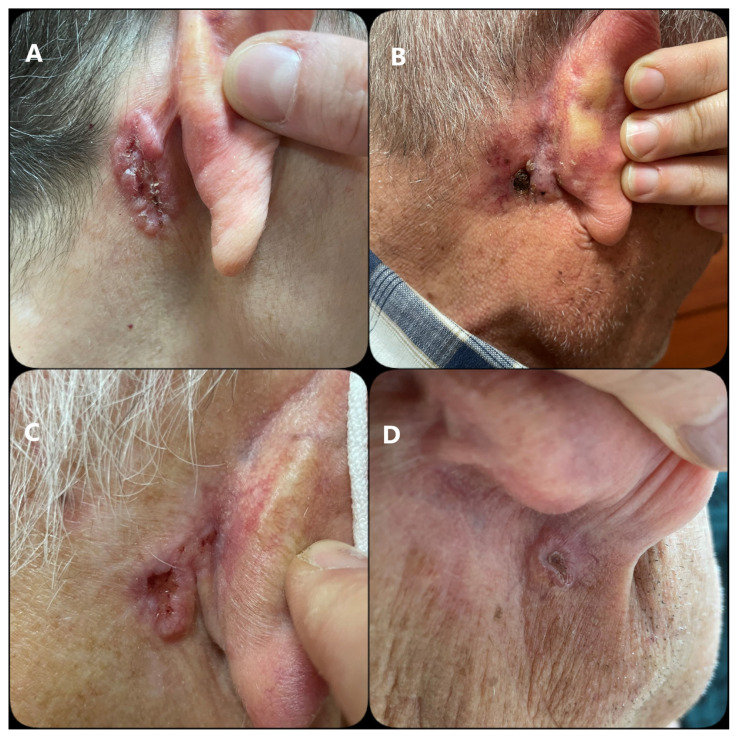
Clinical photographs of the patients. A–C. Infiltrative BCCs in cases 3, 2, and 4, respectively, on the postauricular region, and D. Basosquamous carcinoma on the inferior postauricular sulcus in case 5.

**Table:** Patient characteristics.

Case	Sex	Age (years)	Duration (months)	Pathological subtype	Localization	Linear	Associated risk factors	History of multiple basal cell carcinoma
1	Female	57	4	Superficial	Lateral and posterior part of the auricular lobule	+	History of radiotherapy to the neck	+
2	Male	83	12	Infiltrative	Postauricular area and sulcus	+	-	-
3	Female	62	8	Infiltrative	Postauricular area	+	Use of steroids, methotrexate, and adalimumab	+
4	Male	79	10	Infiltrative	Postauricular area and sulcus	+	History of radiotherapy to the scalp	+
5	Male	75	6	Basosquamous carcinoma	Inferior postauricular sulcus	+	-	+
